# Acupuncture Treatment Associated with Functional Connectivity Changes in Primary Dysmenorrhea: A Resting State fMRI Study

**DOI:** 10.3390/jcm10204731

**Published:** 2021-10-15

**Authors:** Cheng-Hao Tu, Yu-Chen Lee, Ying-Yu Chen, Chun-Ming Chen, Wen-Chi Lu, Yi-Hung Chen, Su-Tso Yang

**Affiliations:** 1 Graduate Institute of Acupuncture Science, China Medical University, Taichung 404333, Taiwan; paulalue@gmail.com (W.-C.L.); yihungchen@mail.cmu.edu.tw (Y.-H.C.); 2 Department of Acupuncture, China Medical University Hospital, Taichung 404332, Taiwan; d5167@mail.cmuh.org.tw; 3 School of Chinese Medicine, China Medical University, Taichung 404333, Taiwan; 4 Department of Chinese Medicine Gynecology, China Medical University Hospital, Taichung 404332, Taiwan; orangedrinkbest@hotmail.com; 5 Department of Medical Imaging, China Medical University Hospital, Taichung 404332, Taiwan; jinmingc@yahoo.com.hk; 6 Traditional Chinese Medicine Research Center, China Medical University, Taichung 404333, Taiwan; 7 Department of Photonics and Communication Engineering, Asia University, Taichung 41354, Taiwan

**Keywords:** primary dysmenorrhea, acupuncture, sanyinjiao, descending pain modulation systems, periaqueductal gray matter, functional connectivity, resting-state functional magnetic resonance imaging

## Abstract

Primary dysmenorrhea (PDM) is the most commonly encountered gynecological problem in reproductive-age women. Acupuncture has been suggested as an effective treatment of PDM that may modulate descending pain modulation systems. In the present study, we used resting-state functional magnetic resonance imaging to investigate possible changes in descending pain modulation systems after acupuncture treatment in women with PDM. Thirty-four right-handed adult women with PDM participated in this randomized, single-blinded, sham-controlled study. Each patient was randomly allocated to an 8-week verum or sham acupuncture intervention on the bilateral Sanyinjiao (SP6). Resting-state functional magnetic resonance imaging was conducted before, during, and after the intervention to measure the spontaneous activity in brain. After the 8-week intervention, both verum and sham groups reported decreased menstrual pain. However, the cessation of decreased functional connectivity (FC) between periaqueductal gray matter and the regions associated with affective pain modulation and attention-related pain modulation were found in the verum but not in the sham group after the 8-week intervention. More decreased FC has been found in the region associated with non-specific effects of acupuncture intervention after the early stage of acupuncture intervention. These results indicated that verum acupuncture may intercept the altered FC in descending pain modulation systems in PDM.

## 1. Introduction

Primary dysmenorrhea (PDM) is the most common gynecological problem among reproductive-age women. Around 10–20% of women experience PDM, causing them to be absent from work or school; thus, PDM affects their daily life [1]. Women with PDM experience cramping pain during menses, which may be due to an abnormal concentration of prostanoids, uterine hypercontractility, or reduced uterine blood flow [2]. Furthermore, they have a lower pain threshold in not only referred pain sites but also the non-referred pain site, indicating altered pain perception in the central nervous system [3,4]. Several abnormal functional and structural changes have been reported in brain regions associated with visceral sensation and pain processing [5,6], as well as how altered functional connectivity (FC) between ventral periaqueductal gray matter (PAG) and other pain-related brain regions in women with PDM indicate the maladaptation of the descending pain modulation system [7].

Acupuncture has been suggested to be an effective treatment for PDM [8]. A recent meta-analysis including 60 randomized control trials reported that manual acupuncture is more effective for menstrual pain relief than no treatment and nonsteroidal anti-inflammatory drugs [9]. Among these 60 trials, the acupoint Sanyinjiao (SP6) was the most frequently used for treating PDM [9]. Acupuncture might alleviate PDM through peripheral or central mechanisms. Peripherally, manual acupuncture on SP6 significantly improves uterine artery blood flow [10]. However, blood prostanoid concentrations were not significantly different in response to electroacupuncture treatment on SP6 or other acupoints [11]. Thus, the treatment effect of acupuncture on PDM may be mediated by vascular response but not changes in blood prostanoid levels.

How acupuncture exerts a central effect on PDM has not been fully explored. Acupuncture on specific acupoints can produce an analgesic effect through descending pain modulation, especially by modulating PAG activity [12]. Furthermore, the termination of chronic pain or of repetitive painful stimuli may be associated with functional and structural resilience changes in the brain [13,14,15]. Therefore, we explored the central mechanisms possibly underlying the effect of acupuncture treatment on PDM. We hypothesized that acupuncture can intercept alterations of FC in the descending pain modulation pathways between PAG and other pain-related brain areas in PDM.

## 2. Materials and Methods

### 2.1. Subjects

This study was conducted in accordance with the Declaration of Helsinki. The protocol was approved by the Institutional Review Board of China Medical University Hospital, Taiwan (CMUH105-REC1-027). All patients were recruited from the internet and then referred to and diagnosed by the Department of Chinese Medicine Gynecology, China Medical University Hospital, Taiwan. All patients received a full explanation of the study and signed written informed consent forms.

The inclusion criteria for subjects were as follows: (a) aged 20–35 years; (b) right-handed; (c) regular menstrual cycle of 27–32 days; and (d) average menstrual pain level in the past 6 months >4 points on a 10-point numerical rating scale. The exclusion criteria were as follows: (a) history of pituitary gland disorder; (b) history of organic pelvic or reproductive system disease(s); (c) history of mental disorder(s), such as major depressive disorder, bipolar disorder, general anxiety disorder, claustrophobia, or schizophrenia; (d) history of brain trauma or neurological disease(s), such as epilepsy, stroke, Parkinson’s disease, or dementia; (e) history of or current pregnancy or plans to become pregnant; (f) presence of metal implants or a pacemaker; (g) use of oral contraceptives, psychiatric medicines, Chinese herbal medicine, or acupuncture treatment in the past 6 months, or taking analgesic drugs 24 h before the magnetic resonance imaging (MRI) scan; and (h) unsuited for acupuncture treatment, as determined by the physicians of the Department of Chinese Medicine Acupuncture, China Medical University Hospital, Taiwan.

### 2.2. Study Design

This study was a randomized, single-blind, sham-controlled study. All subjects were randomly allocated at a 1:1 ratio to the verum or sham acupuncture group by using a computer-generated list of random numbers. During the inception interview, each subject’s general PDM experience in the past was assessed, and handedness was assessed using the Chinese version of the Edinburgh Handedness Inventory [16]. Transabdominal ultrasound was performed to exclude possible organic diseases in the reproductive system that may lead to secondary dysmenorrhea. The resting-state functional MRI (rfMRI) scans were conducted before (pre; on week 0), during (during; on week 4), and after (post; on week 8) an 8-week verum or sham acupuncture intervention session. To reduce the possible interference of menstrual phase, the MRI scans were conducted during days 5–12 of the menstrual cycle. The latest menstrual pain experience and psychological state were also assessed after each MRI scan. Venous blood samples were taken to evaluate the concentrations of estrogen, progesterone, and testosterone within 2 days before or after MRI scans. If the subject had pregnancy concerns, a urine test strip pregnancy test was performed. For ethical reasons, we allowed subjects to take analgesics if their menstrual pain was too severe to bear.

### 2.3. Acupuncture Intervention

All acupuncture interventions were performed by attending physicians who had more than 10 years of acupuncture experience in the outpatient clinics of the Department of Chinese Medicine Acupuncture, China Medical University Hospital, Taiwan. Subjects received a 20-min manual acupuncture intervention on bilateral SP6 twice per week for 8 consecutive weeks (totaling 16 interventions). The acupoint selection was based on a recent meta-analysis study that reported that manual acupuncture is more effective for menstrual pain relief and SP6 was the acupoint most frequently used for treating PDM [9]. The acupoint was cleaned with an alcohol swab, and an O-ring was placed on the acupoint and covered by white surgical tape to conceal the penetration depth of the needle and keep the placebo needle perpendicular to the skin. In the verum acupuncture group, the standard disposable sterile acupuncture needle (diameter: 0.22 mm, length: 30 mm) was used to penetrate vertically into SP6 to a depth of approximately 25 mm. In the sham acupuncture group, the Streitberger placebo needle (Asia-med GmbH. Pullach, Germany), which can effectively serve as the placebo needle in acupuncture [17,18], was used to avoid skin penetration by the blunt-tip placebo needle, which moved into the handle part of the needle when the physician performed puncture action. The movement of the placebo needle into the handle part also effectively blinded subjects by mimicking the phenomenon of shortened needle length after insertion of a needle into the body. In both the verum and sham group, the needle retention time on SP6 was 20 min, and no manipulation of the needles was performed during the retention time. To verify the effectiveness of single blinding, the subjects were asked whether they received verum or sham acupuncture after the 8-week intervention session completed.

### 2.4. Menstrual Pain Experience and Psychological Assessments

The Chinese version of the McGill Pain Questionnaire (MPQ) was used to assess the multidimensional menstrual pain experience [19]. The total score of the pain rating index represents the menstrual pain experience, whereas that of the present pain intensity represents menstrual pain intensity. In addition, the Chinese version of Spielberger’s State-Trait Anxiety Inventory (STAI) and the Chinese version of Beck’s Depression Inventory II (BDI II) were used to assess the level of anxiety and depression, respectively [20,21].

### 2.5. Measurement of Blood Gonadal Hormone Level

Blood concentrations of estrogen, progesterone, and testosterone were assessed using the chemiluminescent immunoassay sandwich method in the Department of Laboratory Medicine, China Medical University Hospital, Taiwan.

### 2.6. Image Acquisition

All brain images were acquired with an 8-channel head coil in a 3.0 Tesla MRI scanner (Signa HDxt, GE Healthcare, Chicago, IL, USA). For rfMRI, four blank scans and 200 resting scans were continuously acquired with an ascending interleaved echo-planar imaging sequence (repetition time = 2500 ms; echo time = 30 ms; flip angle = 90°; matrix = 64 × 64; field of view = 224 × 224 mm^2^; slice number = 40; and slice thickness = 3.5 mm). High-resolution, three-dimensional, T1-weighted anatomical images were also acquired with a spoiled gradient echo sequence (repetition time: 7.356 ms; echo time: 2.736 ms; flip angle = 12°; matrix = 224 × 224 × 170; field of view = 224 × 224 × 170 mm^3^). All scans were acquired in a dimly lit shielding room. Before scanning, subjects were instructed to relax, not to move their head, and keep their eyes opened during the scan.

### 2.7. Image Preprocessing

After discarding blank scans, the rfMRI scans were preprocessed using Statistical Parametric Mapping 12 (SPM12; Wellcome Centre for Human Neuroimaging, University College London, London, UK) with MATLAB 2018a (MathWorks, Sherborn, MA, USA). The rfMRI images were first corrected for slice acquisition times and then realigned to correct the head motions during image scan. Subjects who had head motion larger than 2 mm translation in any x-, y-, z-direction or 2 degree of angular motion were excluded from further image analysis. The T1-weighted anatomical image was co-registered to the mean image generated from the realignment step and then normalized into the Montreal Neurological Institute reference space with a default template using SPM12. The normalization parameters were then applied to all rfMRI images for spatial normalization with the resampled voxel size of 2 × 2 × 2 mm^3^. Finally, the normalized rfMRI images were smoothed with a three-dimensional Gaussian kernel (8-mm full width at half maximum).

The FC maps of PAG and other brain regions were generated by Data Processing and Analysis for Brain Imaging 4.3 with MATLAB 2018a [22]. In preprocessed images, the brain activities in each voxel were linearly detrended and bandpass filtered (0.01–0.08 Hz), and the confounding variables, namely six head movement parameters, the global mean signal, the mean signal of white matter, and the mean signal of the cerebrospinal fluid, were regressed out. The mean time-series activity in the seed region of PAG (defined by an image-based atlas from young adults [23], which is available at https://www.nitrc.org/projects/atag/, accessed on 12 October 2021) was extracted, and voxel-wise correlation analysis was conducted to generate the correlation maps of PAG. Finally, Fisher’s r-to-z transformation was performed to improve the normality of FC maps for statistical analysis.

### 2.8. Statistical Analysis

The statistical analyses regarding demographic data, menstrual pain experience, psychological assessment, and blood gonadal hormone levels were conducted using SPSS v21 (IBM, Armonk, NY, USA). To compare intergroup differences in demographic data and general PDM experience, a two-sample *t* test was conducted. The two-way mixed-model analysis of variance (ANOVA) was used to test the possible interaction effects between groups (verum and sham) and times (before, during, and after) on menstrual pain experience, psychological status, and blood gonadal hormone levels. The significance was set at *p* < 0.05.

The statistical analysis for standardized FC maps of PAG was conducted using SPM12. The flexible factorial design was utilized with group and subject factor as between-subject factor and time factor as within-subject factor. Because our main interest is the effect of intervention was along time, the interaction effect between group and time factor and main effect of all three factors were included in the statistical model. To reveal the treatment-related changes, the difference of differences analysis between groups and times (e.g., verum [post-pre]-sham [post-pre]) were performed using *t*-contrasts in the model. The comparisons between different time-points in each group also were performed using *t*-contrasts in the model to probe possible FC changes that were specifically associated with the intervention. In addition to the factorial analysis, the correlation analysis was also conducted between the menstrual pain experience and FC maps. The latest MPQ scores before each MRI scan were input as a covariate in a separate statistical model, and then centered to overall mean and interacted with group factor that resulted in two separate regressors in the model for verum and sham group, respectively. Considering that fMRI studies often have low statistical power [24], a less stringent significance threshold (cluster-forming threshold as uncorrected voxel *p* < 0.005 with cluster size > 425, corresponding to a family-wise error rate-corrected cluster *p* < 0.1) was applied in the present study. If a significant cluster was found, the representative first eigenvalue among whole cluster in each FC map was extracted without adjustment in SPM to illustrate the FC changes along times in each group.

## 3. Results

### 3.1. Demographic Data and General PDM Experience

**Figure 1:**
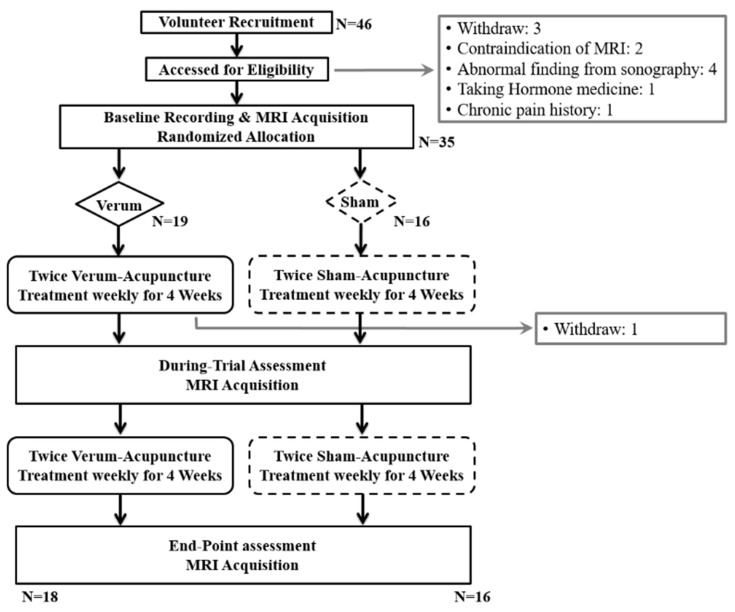
Flowchart of the study.

Forty-six subjects were recruited, but 11 were excluded because of abnormal ultrasonography findings, taking hormone medicine, having a chronic pain history, and withdrawal of consent. Among the 35 subjects included, 19 were allocated to the verum acupuncture group and 16 to the sham acupuncture group. During the intervention session, one subject in the verum group withdrew her consent, leaving 18 patients in the verum group and 16 patients in the sham group, all of whom completed the intervention (Figure 1).

Demographic data were not significantly different between the verum and sham groups in terms of age, handedness, age of menarche, gynecologic age, and length of menstrual cycle. No significant intergroup difference was noted in the age of PDM onset, duration of PDM history, duration of menstrual pain per cycle, menstrual pain experience, and menstrual pain intensity (Table 1). All subjects reported that they believed they were receiving the verum acupuncture intervention. One subject in the sham group reported the use of over-the-counter analgesics during her menstruation. Another subject in the verum group reported the intake of over-the-counter analgesics for a non-menstrual pain condition.

### 3.2. Changes in Menstrual Pain Experience, Psychological Assessment, and Gonadal Hormone Level across Acupuncture Intervention

**Figure 2:**
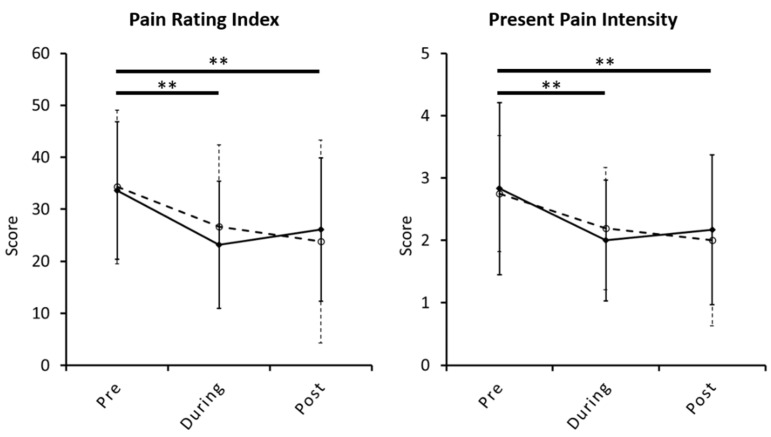
The changes in menstrual pain experience and intensity with verum or sham intervention in patients with primary dysmenorrhea. Both menstrual pain experience and intensity significantly decreased in the early stage of the intervention till the end of intervention session. The solid line and dash line denote verum and sham group, respectively. The double-asterisk mark denotes the significant difference with *p* < 0.01. Data is illustrated as mean ± SD. **, the significant difference with *p* < 0.01.

No significant interaction effect was found on menstrual pain experience or intensity between group and time factors. The main effect analysis revealed no effect on the group factor but a significant effect on the time factor. The *post hoc* analysis (controlled with Sidak correction) revealed that both menstrual pain experience and intensity significantly declined in the early stage of the intervention (weeks 0 to 4) and during the entire intervention (weeks 0 to 8), but no significant change was found in the late stage (weeks 4 to 8) (Figure 2, Table 2). Furthermore, no significant difference was observed in psychological status and gonadal hormone level (Table 2).

### 3.3. Changes in FC Maps across Acupuncture Intervention

Due to a scanner scheduling issue, three and four subjects in the verum and sham groups, respectively, were unable to undergo the rfMRI in week 4. Thus, only 15 and 12 subjects, respectively, were included in the imaging analysis.

**Figure 3:**
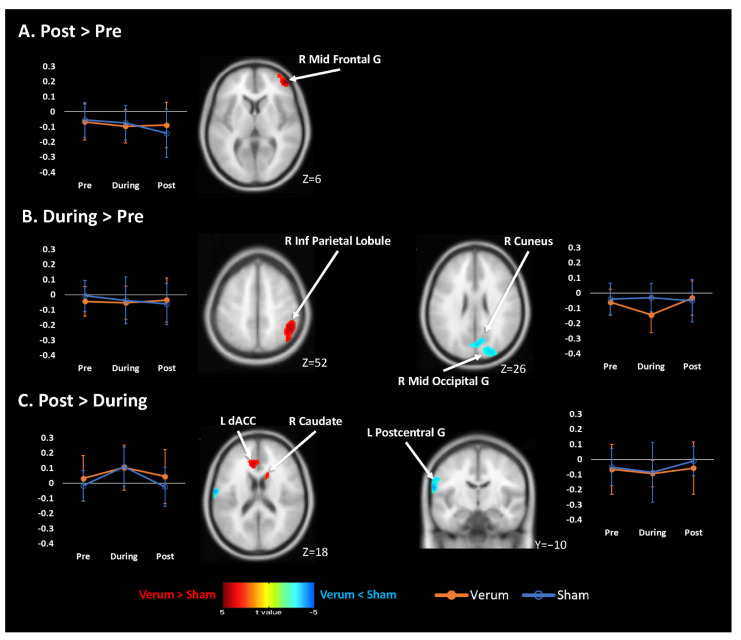
Changes in functional connectivity in descending pain modulation systems between verum and sham acupuncture intervention in patients with primary dysmenorrhea. (**A**) After acupuncture intervention (week 0 to week 8), more increased functional connectivity was found in the right middle frontal gyrus in the verum acupuncture group than in the sham acupuncture group. (**B**) After the early stage of acupuncture intervention (week 0 to week 4), more increased functional connectivity was found in the right inferior parietal lobule in the verum acupuncture group than in the sham acupuncture group, whereas more decreased functional connectivity was found in the right middle occipital gyrus. (**C**) After the late stage of acupuncture intervention (week 4 to week 8), more increased functional connectivity was found in the right dorsal anterior cingulate cortex in the verum acupuncture group than in the sham acupuncture group, whereas more decreased functional connectivity was found in the left postcentral gyrus. Warm and cold colors superimposed on the T1 template denote more increased and more decreased functional connectivity, respectively. The filled circle in orange and the empty circle in blue denote the represented z-value among whole cluster across different time points (expressed in mean ± SD) in the verum group and sham group, respectively. Abbreviation: R, right; L: left; Mid, middle; Inf: inferior; G, gyrus; Lob: lobule; dACC: dorsal anterior cingulate cortex.

After the 8-week intervention, the differences in difference analysis revealed that the verum acupuncture group had significantly higher FC in the right middle frontal gyrus than that of the sham group, whereas no significantly lower FC was found (Figure 3A, Table 3). After extracting the representative z-value among significant cluster, more decreased FC was found in the sham group than in the verum group (Figure 3A, Table S1). There was no significant FC change in either the verum or sham group (Table S2).

Furthermore, after the early stage of intervention (week 0 to week 4), the verum acupuncture group had a significant increase in FC in the right inferior parietal lobule, and a significant decrease in FC in the right middle occipital gyrus when compared to those in the sham group (Figure 3B, Table 3). A significant decrease in FC was found in the sham group in the right inferior parietal lobule when compared to that of the verum group. In the right middle occipital gyrus, FC decreased in the verum group but slightly increased in the sham group (Figure 3B, Table S1). There was a significant increase in FC in the left cerebellum in the verum group after the early stage of intervention, whereas a significant FC decrease was found in the bilateral anterior cingulate cortex (ACC). In the sham group, a significant FC increase was found in the right middle occipital gyrus, whereas no significant FC decrease was found (Table S2).

After the late stage of intervention (week 4 to week 8), the verum group had significant increases in FC in the left dorsal anterior cingulate cortex (dACC) and right caudate nucleus, and a significant decrease in FC in the left postcentral gyrus, when compared to those of the sham group (Figure 3C, Table 3). More decreases in FC were found in the sham group in the left dACC and right caudate nucleus, when compared to those of the verum group. In the left postcentral gyrus, FC increased more in the sham group than in the verum group (Figure 3C, Table S1). There were significant FC increases in bilateral cuneus in the verum group after late stage intervention, whereas a significant decrease in FC was found in the left postcentral gyrus. In the sham group, no significant increase in FC was found, whereas significantly decreased FC was found in the right middle frontal gyrus (Table S2).

For correlation analysis between menstrual pain experience and FC maps of PAG, no significant cluster was found in any brain region.

## 4. Discussion

We conducted this rfMRI study to investigate the possible central mechanisms of acupuncture treatment of PDM in terms of FC changes within the descending pain modulation pathways. Clinically, both verum and sham acupuncture significantly reduced menstrual pain experience and menstrual pain intensity after 8 weeks of intervention. However, more decreased FC was found in the sham group than in the verum group in the regions associated with affective pain modulation and attention-related pain modulation after 8 weeks of intervention, whereas more decreased FC was found in the verum group than in the sham group in the region associated with non-specific effects of acupuncture intervention after the early stage of acupuncture intervention. This implies that the verum acupuncture intervention may intercept the progress of FC changes in descending pain modulation systems in PDM.

Significant interaction across the 8-week verum and sham acupuncture treatments was observed in the right middle frontal gyrus, which corresponds to a portion of ventrolateral prefrontal cortex (vlPFC). The FC in vlPFC gradually decreased in the sham group but was almost unchanged in the verum group. Because the vlPFC may be associated with the modulation of pain-related emotion [25], the decreased FC in the vlPFC may reflect a progressively weakening in affective pain modulation for menstrual pain. Thus, the cessation of decreased FC in vlPFC in the verum group may represent a resilience change of FC in vlPFC to reduce the menstrual pain after verum acupuncture treatment. A similar pattern of FC changes was observed in the right inferior parietal lobule (IPL), a sub-region of the posterior parietal cortex that participates in the lateralized pain-attention network [26,27] and is associated with attention-related pain modulation [28]. Although significant interaction was only found in the early stage of intervention, FC in the sham group gradually decreased across the whole intervention period, whereas FC in verum group remained almost unchanged. Considering the fact that decreased FC in the posterior parietal cortex has been found in PDM when compared to that of the control [7], the FC changes in IPL may reflect progressively reduced attention-related pain modulation for menstrual pain in the sham group, which was restored by verum acupuncture intervention. Thus, verum acupuncture may intercept the progression of altered FC in descending pain modulation systems in PDM.

The FC changes in the middle occipital gyrus after early the intervention stage may be associated with a non-specific effect of acupuncture intervention. Decreased activity in visual-associated middle occipital gyrus (Brodmann area [BA] 19) has been reported when electroacupuncture was applied to visual-related acupoints or non-acupoints [29]. The decreased activity in the middle occipital gyrus was not prevented by administering a local anesthetic to the visual-related acupoint [30]. Moreover, decreased spontaneous activity also has been reported in the middle occipital gyrus after a 4-week acupuncture treatment of patients for migraine without aura [31]. These results suggested a non-specific cross-model inhibition may account for the activity changes in the middle occipital gyrus after acupuncture intervention. On the other hand, a recent study reported that anticipation of placebo analgesic cream, but not control cream, increased activity in the middle occipital gyrus [32]. Hence, the FC changes after early acupuncture intervention may be associated with a non-specific effect acupuncture intervention modulating the neural activity via cross-model inhibition or level of anticipation.

It is noteworthy that there were significant interactions in the dACC and in the left postcentral gyrus in the late stage of intervention. The FC in dACC increased to almost the same level in both verum and sham groups in the early stage of intervention, and decreased in the late stage of intervention with a higher value of FC in the verum group than in the sham group. The dACC has been suggested to be involved in pain processing, which is associated with the survival-related goal [33,34]. The dACC may encode and integrate the nociceptive information to other brain regions [35]. The connectivity between dACC and PAG in terms of mu-receptor binding potential was increased under placebo analgesia [36]. Hence, the FC changes in dACC may reflect the nature of enhanced endogenous opioid activity in the early stage of intervention, with more retained by verum acupuncture. On the other hand, the site of FC changes in postcentral gyrus (BA 43) may represent the visceral region in primary somatosensory cortex and/or secondary somatosensory cortex (SII) and be functionally associated with pain transmission [27]. Previous study reported increased FC in the sensorimotor cortex in PDM patients when compared to that in the control group during their menstrual period [7]. Acupuncture intervention may modulate the FC of sensorimotor regions to other brain regions [37]. Thus, the less changed FC in the verum group than in the sham group may represent the tuning-down effect from acupuncture intervention.

One may argue that the FC changes after acupuncture intervention may be associated with placebo-related mechanisms in the brain. It has been long debated that acupuncture intervention is largely associated with placebo effect. Not only verum but also sham acupuncture intervention significantly relieved menstrual pain in the present study. In addition, it has been reported that PAG, as well as vlPFC, may be engaged with placebo analgesia [38,39]. The vlPFC was activated when patients believed their pain could be controlled [39], while PAG was most activated when a pain cue was present [40]. Since all subjects in the present study believed that they received verum acupuncture intervention, the FC changes across acupuncture intervention may be associated with placebo-related brain mechanisms. However, other brain regions associated with placebo effect (for review, see [41]) had no significant FC change in the present study, even in the analysis between different time points in each group. Moreover, the dynamic FC changes in PAG may not be fully captured in the present study. A recent study reported a significant FC change between vlPFC/dorsolateral prefrontal cortex (dlPFC) and PAG in placebo responder but not in non-responder among chronic back pain patients at the beginning of their first 2-week placebo treatment, and then the FC value was gradually restored at the end of the first placebo treatment and the second 2-week placebo treatment [42]. Considering the timing of the rfMRI scans in our study (week 4 for “during” scans and week 8 for “post” scans), the FC between PAG and other brain regions was not likely to reflect the placebo-associated fast dynamic changes in FC between PAG and other brain regions in the present study. The FC changes in the present study may be associated with treatment-specific mechanisms but not place-related mechanisms in brain.

It should be noted that the FC between PAG and other brain regions was weak, which may lead to the negative finding in the present study. Previous study reported that the temporal signal-to-noise ratio of PAG was lower than other cortical structures [43]. A recent study further demonstrated low FC values between PAG and vlPFC/dlPFC in chronic back pain patients who were on the waiting list for treatment [42]. Hence, the value of FC between PAG and other brain regions may suffer from this noisy signal of PAG that may mask treatment-related changes and lead to the negative finding.

Other limitations of our study should also be noted. First, the number of subjects was relatively small in the present study, which may lower the statistical power. Second, the cluster size for significant threshold was relatively large, which may shadow the FC changes in small structures of the brain. Third, the MPQ is designed for general pain and not specifically for menstrual pain. Fourth, the lack of confirmation of ovulation day and jittering between scan day and blood sampling day in the present study limited the analysis between gonadal hormones and FC changes. Future studies with a larger sample size, specific menstrual pain questionnaire, and confirmed menstrual phase are needed to elucidate the brain mechanisms underlying the effect of acupuncture intervention in PDM.

## 5. Conclusions

In conclusion, our results demonstrated that both verum and sham acupuncture intervention on bilateral SP6 can significantly relieve menstrual pain. However, differences of difference analysis revealed treatment-specific FC changes in descending pain modulation systems. The effect of the verum acupuncture intervention may be underpinned by the intercept of the progressive FC changes in the brain regions that are associated with affective pain modulation, attention-related pain modulation, and pain transmission. The results of this pilot study may be a basis for further discussion of whether acupuncture treatment induces physiological therapeutic alterations or simply induces the placebo effect.

## Figures and Tables

**Table 1:** Clinical and demographic data of women with primary dysmenorrhea.

	Verum (N = 18)	Sham (N = 16)	*p*-Value
Age (year)	24.89 ± 4.59	26.13 ± 4.54	0.44
Headedness (−1~1)	0.84 ± 0.16	0.91 ± 0.17	0.23
The age of menarche (year)	11.72 ± 1.36	11.88 ± 1.36	0.75
Gynecologic age (year)	13.17 ± 4.78	14.25 ± 4.81	0.52
Length of menstrual cycle (day)	31.06 ± 2.34	30.06 ± 1.95	0.19
The age of dysmenorrhea onset (year)	14.50 ± 2.73	14.69 ± 2.77	0.84
Dysmenorrhea history (year)	10.39 ± 5.80	11.44 ± 5.46	0.59
The duration of menstrual pain per cycle (day)	1.89 ± 0.68	1.63 ± 0.62	0.25
MPQ			
PRI (0~78)	35.83 ± 13.28	36.31 ± 12.14	0.91
PPI (0~5)	3.17 ± 1.20	3.25 ± 1.00	0.83

MPQ: McGill Pain Questionnaire; PRI: Pain Rating Index; PPI: Present Pain Intensity. All values are presented as mean ± SD.

**Table 2:** Menstrual pain experience, psychological assessment, and gonadal hormone level across acupuncture intervention.

	Verum (N = 18)			Sham (N = 16)			*p*-Value		
	Pre	During	Post	Pre	During	Post	Interaction	Group	Time
MPQ									
PRI total (0~78)	33.61 ± 13.22	23.17 ± 12.20	26.11 ± 13.79	34.31 ± 14.78	26.69 ± 15.70	23.81 ± 19.46	0.52	0.88	0.001 ^#&^
PPI (0~5)	2.83 ± 1.38	2.00 ± 0.97	2.17 ± 1.20	2.75 ± 0.93	2.19 ± 0.98	2.00 ± 1.37	0.77	0.94	0.009 ^#&^
BDI	8.11 ± 6.33	7.89 ± 5.80	7.72 ± 9.41	10.69 ± 9.51	6.38 ± 3.65	7.63 ± 5.77	0.32	0.86	0.23
STAI									
State (20~80)	43.17 ± 8.96	41.67 ± 7.30	40.78 ± 10.94	43.38 ± 9.98	43.13 ± 8.88	40.81 ± 9.30	0.89	0.83	0.29
Trait (20~80)	47.33 ± 7.22	45.61 ± 4.05	45.72 ± 10.01	46.94 ± 9.77	45.06 ± 8.95	43.56 ± 9.63	0.76	0.68	0.15
Hormones									
Estradiol (pg/mL)	87.89 ± 73.90	63.44 ± 30.63	116.28 ± 108.75	77.69 ± 57.44	94.63 ± 63.10	117.44 ± 81.97	0.45	0.65	0.05
Progesterone (ng/mL)	2.77 ± 4.47	0.75 ± 1.29	2.67 ± 5.11	1.23 ± 2.61	2.26 ± 4.70	2.77 ± 4.12	0.24	0.98	0.40
Testosterone (ng/mL)	0.52 ± 0.28	0.58 ± 0.28	0.64 ± 0.27	0.51 ± 0.15	0.49 ± 0.15	0.51 ± 0.41	0.05	0.32	0.05

Pre: pre-treatment; During: during-treatment; Post: post-treatment; MPQ: McGill Pain Questionnaire; BDI: Beck’s Depression Inventory; STAI: State-Trait Anxiety Inventory; PRI: Pain Rating Index; PPI: Present Pain Intensity; ^#^: Significant difference between weeks 0 and 4; ^&^: Significant difference between weeks 0 and 8. All values are presented as mean ± SD.

**Table 3:** Changes in functional connectivity map between verum and sham acupuncture intervention.

Verum > Sham							Verum < Sham						
				Coordinate							Coordinate		
Anatomical Area	BA	Size	t-max	x	y	z	Anatomical Area	BA	Size	t-max	x	y	z
Post > Pre													
R Mid Frontal G	46/10	622	4.71	50	50	6	n.s.						
During > Pre													
R Inf Parietal Lob	40/39/7	1030	4.12	48	−46	52	R Mid Occipital G	19	1153	4.23	32	−88	14
							R Cuneus	18		3.55	4	−76	26
Post > During													
L dACC	24	433	3.92	−8	32	16	L Postcentral G	43	483	4.16	−64	−8	20
R Caudate N			3.22	14	16	18	L Precentral G	6		3.86	−60	−4	30
							L Sup Temporal G	42		3.42	−68	−22	10

BA: Brodmann area; Size: number of voxels in the cluster; t-max: peak t value; Pre: pre-treatment; During: during-treatment; Post: post-treatment; L: left; R: right; Sup: superior; Inf: inferior; Mid: middle; dACC: dorsal anterior cingulate cortex; G: gyrus; Lob: lobule; N: nucleus; n.s.: non-significant.

## Data Availability

All data analyzed in this study are available from the corresponding author on reasonable request.

## Supplementary Materials

The following are available online at https://www.mdpi.com/article/10.3390/jcm10204731/s1, Table S1: Represented z-value among significant clusters between verum and sham acupuncture intervention, Table S2: Changes in functional connectivity map after verum or sham acupuncture intervention.
