# Supplementary material for: Acupuncture Treatment Associated with Functional Connectivity Changes in Primary Dysmenorrhea: A Resting State fMRI Study
Source: J Clin Med. 2021 Oct 15;10(20):4731. doi: 10.3390/jcm10204731 (PMC8537009; doi:10.3390/jcm10204731)
Supplement: Supplementary file 1 [file jcm-10-04731-s001.zip › jcm-1373966-supplementary.pdf]

Supplementary Material

1 Supplementary Tables

1.1 Supplementary Table S1. Represented z-value among significant cluster between verum and sham acupuncture intervention

	Verum (N=15)			Sham(N=12)		
	Pre	During	Post	Pre	During	Post
**Post vs Pre**						
R Mid Frontal G	-0.07±0.12	-0.10±0.11	-0.09±0.15	-0.05±0.12	-0.07±0.11	-0.14±0.16
**During vs Pre**						
R Inf Parietal Lob	-0.04±0.10	-0.05±0.11	-0.03±0.15	-0.01±0.10	-0.04±0.15	-0.06±0.14
R Mid Occipital G	-0.06±0.09	-0.14±0.12	-0.03±0.11	-0.04±0.10	-0.03±0.09	-0.05±0.14
**Post vs During**						
L dACC	0.03±0.15	0.10±0.15	0.04±0.18	-0.02±0.10	0.11±0.13	-0.02±0.13
L Postcentral G	-0.01±0.14	0.01±0.10	0.01±0.18	0.05±0.23	-0.08±0.29	0.03±0.12

Pre: pre-treatment; During: during-treatment; Post: post-treatment; L: left; R: right; Mid: middle; dACC: dorsal anterior cingulate cortex; G: gyrus; Lob: lobule. All values are presented as mean ± SD.

1.2 Supplementary Table S2. Changes in functional connectivity map after verum or sham acupuncture intervention

**Verum**							**Sham**						
				Coordinate							Coordinate		
Anatomical Area	BA	Size	t-max	x	y	z	Anatomical Area	BA	Size	t-max	x	y	z
**Post > Pre**													
n.s.							n.s.						
**Post < Pre**													
n.s.							n.s						
**During > Pre**													
L Cerebellum	Pyramis	441	5.11	-34	-76	-50	R Mid Occipital G	19	981	4.85	32	-88	16
							R Cuneus	18		4.17	16	-94	20
**During < Pre**													
Bi ACC	24	534	4.19	-2	22	-2	n.s.						
			3.66	-2	30	2							
R Inf Frontal G	47		3.35	30	18	-14							
**Post > During**													

R Cuneus	19/18	503	3.55	2	-78	32	n.s.						
L Precuneus	19		3.24	-8	-82	44							
**Post < During**													
L Postcentral G	43	595	4.21	-64	-8	20	R Mid Frontal G	46	429	3.71	48	50	6
L Sup Temporal G	42		3.87	-66	-26	14	R Sup Frontal G	10		3.59	24	46	18
L Mid Frontal G	9		3.81	-54	18	36							

BA: Brodmann area; Size: number of voxels in the cluster; t-max: peak t value; Pre: pre-treatment; During: during-treatment; Post: post-treatment; L: left; R: right; Bil: bilateral; Sup: superior; Mid: middle; Inf: inferior; ACC: anterior cingulate cortex; G: gyrus; Lob: lobule; n.s.: non-significant.
